# Highlighting the limitations of static microplate biofilm assays for industrial biocide effectiveness compared to dynamic flow conditions

**DOI:** 10.1111/1758-2229.13214

**Published:** 2023-11-27

**Authors:** Kyle B. Klopper, Elanna Bester, Martha van Schalkwyk, Gideon M. Wolfaardt

**Affiliations:** ^1^ Department of Microbiology Stellenbosch University Stellenbosch South Africa; ^2^ Department of Chemistry and Biology Toronto Metropolitan University Toronto Ontario Canada

## Abstract

The minimal inhibitory concentration of an antimicrobial required to inhibit the growth of planktonic populations (minimum inhibitory concentration [MIC]) remains the ‘gold standard’ even though biofilms are acknowledged to be recalcitrant to concentrations that greatly exceed the MIC. As a result, most studies focus on biofilm tolerance to high antimicrobial concentrations, whereas the effect of environmentally relevant sub‐MIC on biofilms is neglected. The effect of the MIC and sub‐MIC of an isothiazolinone biocide on a microbial community isolated from an industrial cooling system was assessed under static and flow conditions. The differential response of planktonic and sessile populations to these biocide concentrations was discerned by modifying the broth microdilution assay. However, the end‐point analysis of biofilms cultivated in static microplates obscured the effect of sub‐MIC and MIC on biofilms. A transition from batch to the continuous flow system revealed a more nuanced response of biofilms to these biocide concentrations, where biofilm‐derived planktonic cell production was maintained despite an increase in the frequency and extent of biofilm sloughing. A holistic, ‘best of both worlds’ approach that combines the use of static and continuous flow systems is useful to investigate the potential for the development of persistent biofilms under conditions where exposure to sub‐MIC and MIC may occur.

## Graphical abstract

The nature of broth microdilution and crystal violet biofilm assays facilitates high‐throughput screening under static conditions, but lacks the capacity to inform on the complexity of biofilms that develop under dynamic, continuous flow conditions. The combined CEMS‐BioSpec system allows for the real‐time monitoring of biofilm activity and biomass in response to antimicrobial exposure. The results demonstrate the utility of a biocide evaluation pipeline that incorporates the best of both static and dynamic biofilm systems to evaluate the effect of industrial biocides on multispecies communities at sub‐MIC and MIC and above.

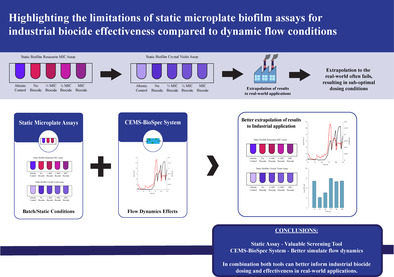

## INTRODUCTION

Industrial microbiologists and process engineers largely agree on the juxtaposition that microbial biofilms can be either beneficial (bioremediation) or deleterious (biofouling), depending on the environment in question. The role of biofilms as a predominant mode of microbial growth has gradually been accepted by the broader scientific and engineering community (O'Toole, 2010; Widder et al., 2016). Microbial biofilms are broadly defined as complex communities of microbial cells embedded in an extracellular matrix attached to a surface (Giaouris et al., 2015; Kwasny & Opperman, 2010; Narenkumar et al., 2017), whereas biofouling is the undesired colonisation of solid–liquid interfaces (Dobosz et al., 2015; Flemming, 2011; Rao, 2015). Biofouling mitigation strategies are predominately based on a chemical‐centric approach, with biocide application to control biofouling being a well‐established antifouling approach in various water cooling systems (Dobosz et al., 2015; Flemming, 2011; Rao, 2015; Silva et al., 2020). Biocides are active chemicals (oxidising and non‐oxidising) or biological agents used to exert control over unwanted microbial populations (sessile and planktonic) through growth inhibition (microbiostatic), killing (microbiocidal) and/or removal (dispersion) (Flemming, 2011; Karsa, 2007; Michalak & Chojnacka, 2014; Rao, 2015; Silva et al., 2020). Biocide activity is governed by the concentration of the agent, exposure time, temperature, pH and organic load (Javaherdashti & Akvan, 2020; Karsa, 2007; Michalak & Chojnacka, 2014; Silva et al., 2020). Isothiazole and its derivatives, such as isothiazolinone are some of the most extensively used biocides in industrial cooling circuits for the treatment and management of biofouling (Karsa, 2007; Silva et al., 2020). The stability of isothiazolinone across a wide range of environmental conditions (temperature, pH, etc.) and its broad spectrum of activity at low concentrations make it a reliable and economical biocide (Karsa, 2007; Silva et al., 2020). Since it is a non‐oxidising, electrophilic biocide, it arrests microbial growth initially (within a few minutes), and extended exposure results in cell death (a few hours) (Silva et al., 2020).

The minimum inhibitory concentration (MIC) is generally accepted as the ‘gold standard’ for the determination of microbial susceptibility to a specific antimicrobial agent (Andrews, 2001; Teh et al., 2017; Wiegand et al., 2008). The MIC can be defined as the lowest concentration of an antimicrobial agent that will inhibit the visible growth of a microorganism after incubation under specific conditions (Andrews, 2001; Wiegand et al., 2008). Turbidimetric measurements form the basis of conventional MIC assays, making them prone to interference by sedimentation of organisms, poor solubility of test compounds, opaqueness of compounds and so forth (Andrews, 2001; Chakansin et al., 2022; Elshikh et al., 2016; Wiegand et al., 2008). To overcome some of these limitations, the blue metabolic indicator resazurin has been included in a modified MIC assay (Chakansin et al., 2022; Elshikh et al., 2016; Mann & Markham, 1998; Teh et al., 2017). The use of resazurin as a metabolic indicator relies on the ability of viable cells to irreversibly reduce resazurin (blue) to resorufin (pink) (Chakansin et al., 2022; Elshikh et al., 2016; Mann & Markham, 1998; Teh et al., 2017). The inclusion of the metabolic indicator provides a simple, sensitive, robust and high‐throughput assay for determining the antimicrobial activity of various compounds without the limitations associated with the standard MIC broth microdilution assay (Chakansin et al., 2022; Elshikh et al., 2016; Mann & Markham, 1998).

MIC determinations provide a starting point for the evaluation of a biocide's antimicrobial activity. However, it does not reveal the inhibitory effect on biofilms. Due to the inherent recalcitrant nature of biofilms, the ability of biocides to inhibit or disperse biofilms is of critical importance for mitigating the impact of biofouling (Dobosz et al., 2015; Kwasny & Opperman, 2010; Narenkumar et al., 2017). The most broadly adopted assay for the evaluation of antibiofilm activity of antimicrobials are static biofilm assays (various permutations of the 96‐well crystal violet [CV] assays) (Davies et al., 1998; Kim & Park, 2013; Kwasny & Opperman, 2010; Mendoza‐Olazarán et al., 2015; Merritt et al., 2005; Narenkumar et al., 2017; O'Toole, 2010; Rajamani et al., 2019). These assays are popular due to their simplicity, cost‐effectiveness, flexibility, wide application and high‐throughput nature (Kwasny & Opperman, 2010; Merritt et al., 2005; O'Toole, 2010).

While static biofilm assays are efficient tools for the evaluation of new and existing biocides for potential antimicrobial and dispersant properties, these assays are limited to the evaluation of the initial stages of biofilm formation (Kwasny & Opperman, 2010; Merritt et al., 2005; O'Toole, 2010). The evaluation of mature biofilms and their stochastic nature can only be achieved through the introduction of bulk‐liquid flow, representing industrial systems typically affected by biofouling. The dynamic nature of mature biofilms furthermore requires continuous, high‐frequency sampling/measurement conditions to facilitate a greater understanding of biofilm responses to antimicrobial agents. However, most continuous flow methodologies only provide a temporal/periodic and destructive view into biofilm development, structures and survival (Adair et al., 2000; O'Toole, 2010; Sønderholm et al., 2018).

The evaluation of new and existing biocides for use in the mitigation/control of biofouling within an industrial setting is as nuanced and complex as the biofilms they are designed to treat. Therefore, a holistic approach is required that incorporates various techniques for effective monitoring of biofilms. These include using the high‐throughput, scalable, simple, cost‐effective screening tool provided by static assays in concert with the time‐consuming, complex, but more realistic results obtained from continuous flow methodologies. Here, we report on a biocide evaluation pipeline that incorporates the best of both static and dynamic biofilm systems for evaluating the effect of a widely used industrial biocide, isothiazolinone, at sub‐MIC and MIC on multispecies biofilm communities. The combined approach yielded valuable insights into the limitations and advantages of both approaches.

## EXPERIMENTAL PROCEDURES

### Microorganisms and cultivation conditions

Two *Pseudomonas* strains were included as controls to compare biocide susceptibility, namely, *Pseudomonas fluorescens* CT07 previously isolated from a cooling tower (Bester et al., 2005), and *Pseudomonas aeruginosa* PA01, a widely studied Gram‐negative biofilm‐forming organism. The control strains were preserved in 40% v/v glycerol at −80°C.

An environmentally representative microbial community, previously exposed to an isothiazolinone‐based biocide, was obtained from the sump water of an industrial cooling tower. Upon receipt of the water samples, it was aliquoted into cryovials with sterile glycerol (final concentration 40% v/v) and preserved at −80°C.

The plant provided the authors with access to the routinely measured chemical, microbial, and physical parameters of the cooling water, under the condition of non‐disclosure. This data informed the selection and composition of the growth medium used in this study and indicated that the sump microbial community was dominated by *Pseudomonas* spp., which was confirmed by culturing and molecular characterization. Briefly, the water samples were filtered through a 0.45‐μm pore‐size GN‐6 Metricel grid membrane filter (Pall Corporation) using the membrane filtering technique according to ISO 16266:2006(E) (ISO Pseudomonas, 2006). The membrane filters were placed on *Pseudomonas* CF and CNC selective agar (Oxoid Ltd., Hampshire, UK). Individual isolates from the membranes were sub‐cultured on the selective media before inoculating pure cultures into 3 g L^−1^ Tryptic soy broth for DNA extraction. DNA was extracted using the Zymo *Quick*‐DNA fungal/bacterial kit (Zymo, Irvine, CA, USA) according to the manufacturer's recommendations. Molecular identification was achieved using 16S rRNA PCR amplification (Klopper et al., 2018) and sequencing of the PCR products (Central Analytical Facility, Stellenbosch University).

To simulate industrial cooling waters, whilst standardising the composition of the growth medium across experimental conditions, the microbial community and control strains were cultivated in synthetic cooling water (SCW) (MacDonald et al., 2000) with slight modifications (mSCW). The modifications included an adjustment in the concentration of yeast extract from 10 to 100 mg L^−1^ to increase nitrogen and vitamin availability for consistent growth. The CaCO_3_ (250 mg L^−1^) was omitted due to poor water solubility (13 mg L^−1^) to prevent precipitate from clogging the experimental system and interfering with absorbance measurements. The pH of mSCW was 8.0, which corresponded well with the average pH of cooling waters reported in the literature (pH of 8.03 ± 0.5) (Iervolino et al., 2017; Paranjape et al., 2020; Pinel et al., 2020) and with the average pH of the sump water from which the sump microbial community was isolated. All experimentation was conducted at 22°C (close to ambient temperature, minimal heat/cooling required) due to the average temperature of cooling water in literature being 23 ± 3.8°C (Adams et al., 1978; Iervolino et al., 2017; Paranjape et al., 2020; Pinel et al., 2020).

All precultures were incubated at 22°C for 18–24 h and standardised to an optical density of 0.1 at 595 nm (OD_595m_, corresponding to approximately 1.0 × 10^8^ CFU mL^−1^) in sterile mSCW before commencement of experimentation, unless stated otherwise.

All reagents used were from Sigma‐Aldrich (South Africa) unless stated otherwise.

### Industrial isothiazolinone biocide formulation and usage

An industrial/commercial isothiazolinone biocide consisting of a 3:1 mixture of 5‐chloro‐2‐methyl‐1,2‐thiazol‐one (CMIT) and 2‐methyl‐4‐isothiazolin‐3‐one (MIT) was used in all experiments. A working stock (160 mg L^−1^) of the biocide was freshly prepared in sterile mSCW before each experiment and used within 24 h for all of the experimentation, except the biocide stability test. All experimentation was conducted using the same batch of isothiazolinone biocide.

### Resazurin‐based MIC determinations

A resazurin‐based assay was used to determine the MIC of the isothiazolinone biocide for the sump community by modifying the traditional broth microdilution assay (Chakansin et al., 2022; Elshikh et al., 2016; Foerster et al., 2017; Teh et al., 2017). Pure cultures of *P. fluorescens* CT07 and *P. aeruginosa* PA01 were included in the assays as references. A resazurin stock solution was prepared at a concentration of 0.1 mg mL^−1^ (Sigma‐Aldrich, South Africa) in phosphate‐buffered saline (pH 7.2), vortexed, filter sterilised (0.22‐μm pore‐size filter) and stored in a dark container at 4°C until required.

The resazurin‐MIC (RMIC) assays were performed in sterile 96‐well round‐bottom polystyrene microplates for high‐throughput screening. A water reservoir was created in the plate by pipetting 250 μL into each well around the perimeter of the plate, to minimise the influence of evaporation on test wells. Each microbial preculture was standardised to an OD_595nm_ of 0.2 (double the final OD_595nm_) in fresh mSCW. The working stock of the isothiazolinone biocide was diluted in mSCW at twice the desired starting concentration (160 mg mL^−1^). The range of biocide concentrations to be tested was prepared using doubling dilutions (80 mg L^−1^ to 0.31 mg L^−1^ final concentration per well), where 80 mg L^−1^ represents the maximum manufacturer‐recommended slug/shot concentration for treatment of biofouling in cooling circuits. Thereafter, 100 μL of the preculture, standardised in mSCW was added to the wells containing mSCW‐diluted biocide (100 μL) and the control wells (containing only 100 μL mSCW), resulting in approximately 1.0 × 10^8^ CFU mL^−1^ per well (final volume of 200 μL). Uninoculated wells (negative control) containing only biocide and mSCW across the same concentration range were included to account for any influence of the biocide on the reduction of resazurin. No colour change was observed for these wells during experimentation.

The inoculated microplates were incubated for 24 h at 22°C (without agitation). After incubation, resazurin was added to all wells (20 μL per well, final concentration of 0.01 mg mL^−1^), followed by an 8‐h incubation at 22°C to allow for the reduction of resazurin from blue to purple/pink. The colourimetric change was measured at 570 nm (absorbance peak of blue resazurin, oxidised form) and 600 nm (absorbance peak of pink resorufin, reduced form), using 600 nm as a reference wavelength with an iMark™ microplate reader (Bio‐Rad, Lasec, South Africa). All data was normalised to the 600 nm value. The percentage reduction of resazurin was calculated in accordance with established protocols and equations (Bio‐Rad, n.d.; Lee & Jain, 2017). No visible colour change corresponds to a low percentage of resazurin reduction (measured and calculated) and is indicative of little or no metabolic activity. The RMIC assays were performed in biological duplicates with three replicate plates each.

The nature of microplate assays—whether conventional microbroth dilution MIC assays rely on turbidity measurements or the RMIC assay which relies on metabolic activity—examine the influence of an antimicrobial on the contents of each well, which likely consists of both planktonic as well as sessile cell populations. To determine the MIC for the planktonic and sessile constituents of the population, the same procedure was followed as the RMIC assay, with slight modification post the initial 24‐h incubation to separate the planktonic from the attached population of cells. The planktonic population in each well was separated from the sessile population by pipetting the aqueous fractions containing planktonic cells, spent growth medium and biocide (±180 μL) into a new microplate and adding 20 μL fresh mSCW to yield a final volume of 200 μL per well. The wells containing the sessile populations were filled with 200 μL of fresh mSCW. Resazurin was added (20 μL) to all the wells of both the planktonic and sessile microplates and incubated at 22°C for 8 h to observe any resulting colour change. Measurements and data processing were completed as described above.

During the process of separating the planktonic population from the sessile population in the wells, the planktonic portion contained both cells and spent growth medium and biocide. However, the planktonic cells could not be separated from the spent growth medium and biocide without potentially causing damage or changes to the planktonic cells (e.g., by using centrifugation), which could influence experimental outcomes. Therefore, the same volume of fresh mSCW could not be added to the planktonic populations (180 μL fresh mSCW for sessile vs. 20 μL for planktonic populations).

### Static CV microplate biofilm assays

Biofilm biomass measurements using CV were performed in sterile 96‐well round‐bottom polystyrene microplates in accordance with previous research (Cruz et al., 2018; Kwasny & Opperman, 2010; Merritt et al., 2005; Narenkumar et al., 2017; O'Toole, 2010; Rajamani et al., 2019) with slight modifications. Briefly, the same procedure for the preparation and inoculation of the microplate was followed for the RMIC assay. The same biocide concentration range was generated using doubling dilutions. After incubation, the planktonic/aqueous proportion from each well was removed and discarded. The wells were washed twice with saline (0.9% m/v NaCl) before adding 200 μL of 0.01% w/v CV solution to each well (control and test wells) and incubating at room temperature for 30 min. The unbound CV was discarded, and the plates were rinsed twice with 200 μL dH_2_O per well. The plates were inverted and dried at room temperature for 30 min. Once dry, 200 μL of 30% (v/v) acetic acid was added to each well and incubated for 15 min at room temperature. Complete solubilisation of the CV bound to the biomass was achieved by vigorous pipetting. Thereafter, 125 μL of the solubilised CV was transferred to a new 96‐well flat‐bottom polystyrene microplate, and absorbance was measured at 570 nm using the iMark™ microplate reader (Bio‐Rad, Lasec, South Africa). The experiment was performed in biological duplicate with three replicate plates per experiment.

In addition, the ability of the isothiazolinone to remove 24‐h‐old biofilms was assessed using CV staining of biomass attached to the wells of microplates (Davies et al., 1998; Kim & Park, 2013; Mendoza‐Olazarán et al., 2015). The assay was conducted by pipetting 100 μL of mSCW without biocide and 100 μL of standardised cultures (OD_595nm_ of 0.2) into the wells of a 96‐well round‐bottom polystyrene microplate. The plates were incubated for 24 h at 22°C. The planktonic/aqueous portion was aspirated from each well and discarded. The plates were rinsed twice with sterile saline and filled with 100 μL fresh mSCW and 100 μL of the same concentration range of the biocide as previously used for the RMIC assay. The plates were incubated for 24 h at 22°C before removing and discarding the aqueous phase. The wells were filled with 200 μL of CV (0.01% m/v) and incubated at room temperature for 30 min. The unbound CV was removed and discarded, and plates were rinsed twice with 200 μL dH_2_O. The CV was solubilised and analysed as described previously. The experiment was performed in duplicate with three replicate plates each.

### Biocide stability assay

To ensure that mSCW had minimal influence on the stability of the biocide, a stability assay was conducted. The stability of isothiazolinone was assessed over 5 days to determine its efficiency once diluted in mSCW for preparation of the working stock. The same experimental procedures were followed for the RMIC assays with slight modifications. Briefly, a working stock (160 mg L^−1^) was prepared and stored in a cool, dark place until required (96 h in total). An RMIC assay for the sump microbial community was performed at 24, 72, and 96 h after preparation of the working stock to determine whether the minimal inhibitory concentration of the biocide changed due to a loss of activity. This was performed in duplicate with three replicate microplates during each experiment.

### In situ biofilm biomass and metabolic activity monitoring under continuous flow conditions

**FIGURE 1:**
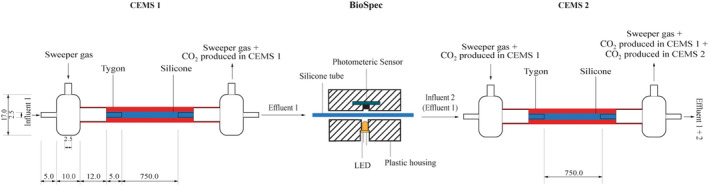
Schematic of the CEMS‐BioSpec system used for the real‐time monitoring of biofilm parameters under continuous flow conditions. The CO_2_‐free sweeper gas is introduced under controlled flow into the annular space of CEMS (red‐shaded region), allowing for the collection of biofilm‐evolved CO_2_ and subsequent analysis by a downstream infrared CO_2_ analyser. Biofilm biomass was measured between the two CEMS units (BioSpec) via the internal silicone tube (blue‐shaded region, containing biofilm biomass) being passed through a cavity with an LED illuminating the tube from one side and a digital light sensor on the transverse side measuring the amount of illumination absorbed by biomass in the tube. All dimensions are to the nearest mm (Klopper et al., 2020).

In situ biofilm biomass and metabolic activity measurements were conducted in real‐time under continuous flow conditions using the combined CEMS‐BioSpec system previously developed in our group (Klopper et al., 2020). Briefly, the system consists of two carbon dioxide evolution measurement systems (CEMS) (Bester et al., 2010; Klopper et al., 2020; Kroukamp & Wolfaardt, 2009), each comprised of a silicone tube as a continuous flow bioreactor encased in an outer Tygon tube. The Tygon tube facilitates the removal of microbially‐derived CO_2_ by a sweeper gas and subsequent analysis by a CO_2_ analyser (Figure 1). This system is premised on the high CO_2_ permeability of silicone (permeability coefficient of 20,132) vs the near‐impermeability of Tygon tubing (permeability coefficient of 270). The BioSpec (Klopper et al., 2020) component of the CEMS‐BioSpec system is based on the well‐established use of light absorbance/transmission/scattering to measure microbial biomass. Briefly, BioSpec was located in between the two CEMS and consisted of silicone tubing, sandwiched perpendicularly between an amber LED (595 _nm_) and a high‐accuracy digital light sensor (Figure 1).

The CEMS‐BioSpec system was connected to a sterile growth medium reservoir, glass manifold/bubble trap, and a Watson Marlow 205S peristaltic pump using silicone tubing (upstream of the system) and a waste container (downstream of the system). The silicone tubing was disinfected in place with 3.5% (v/v) sodium hypochlorite for 2 h and then rinsed with sterile distilled water overnight, with flow provided by the peristaltic pump. The water was displaced from the system with sterile growth medium (mSCW) (0 mg L^−1^ biocide) only, or with the biocide (final concentration of 0.31, 0.62, 1.25 or 2.50 mg L^−1^ biocide), before inoculation. The system was inoculated with 1 mL of a sump microbial community preculture (standardised at an OD_595nm_ of 0.1), with the flow ceased for 30 min to allow for colonisation of the tubing. The flow of the growth medium was initiated at a rate of 12.5 mL h^−1^ (clearance of system volume every 15 min) with the temperature controlled at 22°C using a water bath. CEMS‐Biospec measurements of whole‐biofilm metabolic activity (CO_2_ production) and biomass accumulation (absorbance) were logged automatically at 1‐min intervals throughout each experimental run.

### Biofilm‐derived planktonic cell yield under continuous flow conditions

The concentration of planktonic cells derived from the biofilm was determined at 24‐h intervals. Briefly, the effluent was collected from the CEMS‐BioSpec system, serially diluted in Dey‐Engley neutralising broth (BD, South Africa), and plated on mSCW containing 1.5% (m/v) agar. Plates were incubated for 24 h at 22°C before counting the number of colonies. Biofilm cultivation was performed in duplicate and plate counts were done in triplicate for each biocide concentration.

### Biofilm development in the presence of sub‐MIC, MIC and double MIC of the biocide

The CEMS‐BioSpec system was prepared and inoculated as previously stated, except for the inclusion of biocide in the growth medium reservoir. Biocide was added to the mSCW‐containing reservoir at the following final concentrations: 0, 0.31 (quarter MIC), 0.62 (half MIC), 1.25 (MIC) and 2.5 mg L^−1^ (double MIC) for the entire duration of these experiments. The biocide‐containing growth medium was introduced into the system using the peristaltic pump. The system was inoculated as described previously and the flow of growth medium containing biocide was initiated at a flow rate of 12.5 mL h^−1^ with the temperature controlled at 22°C using a water bath for the duration of the experiment.

### Abiotic controls for continuous flow biofilm exposure to the biocide

The abiotic response of the CEMS‐BioSpec system to the mSCW alone and mSCW with various concentrations of biocide (0.31, 0.62 and 1.25 mg L^−1^) was assessed to determine the extent to which the added biocide contributed to changes in CO_2_ evolution from the mSCW. In essence, the same experimental setup was followed as above with the introduction of sterile mSCW into the system. The flow of medium/medium with biocide was maintained until a steady state was achieved for both the BioSpec absorbance and CEMS CO_2_ evolution rate measurements. This was followed by the sequential introduction of mSCW containing the increasing concentrations of biocide. Finally, mSCW was reintroduced before the termination of the experiment.

### Statistical analysis

Analysis of variance with Tukey post hoc tests was conducted for the microplate datasets utilising the IBM SPSS 22 software package (*p* < 0.05). Where appropriate, all vertical error bars represent standard deviation, and sample sizes are indicated in parentheses.

## RESULTS

### Resazurin‐based MIC

**FIGURE 2:**
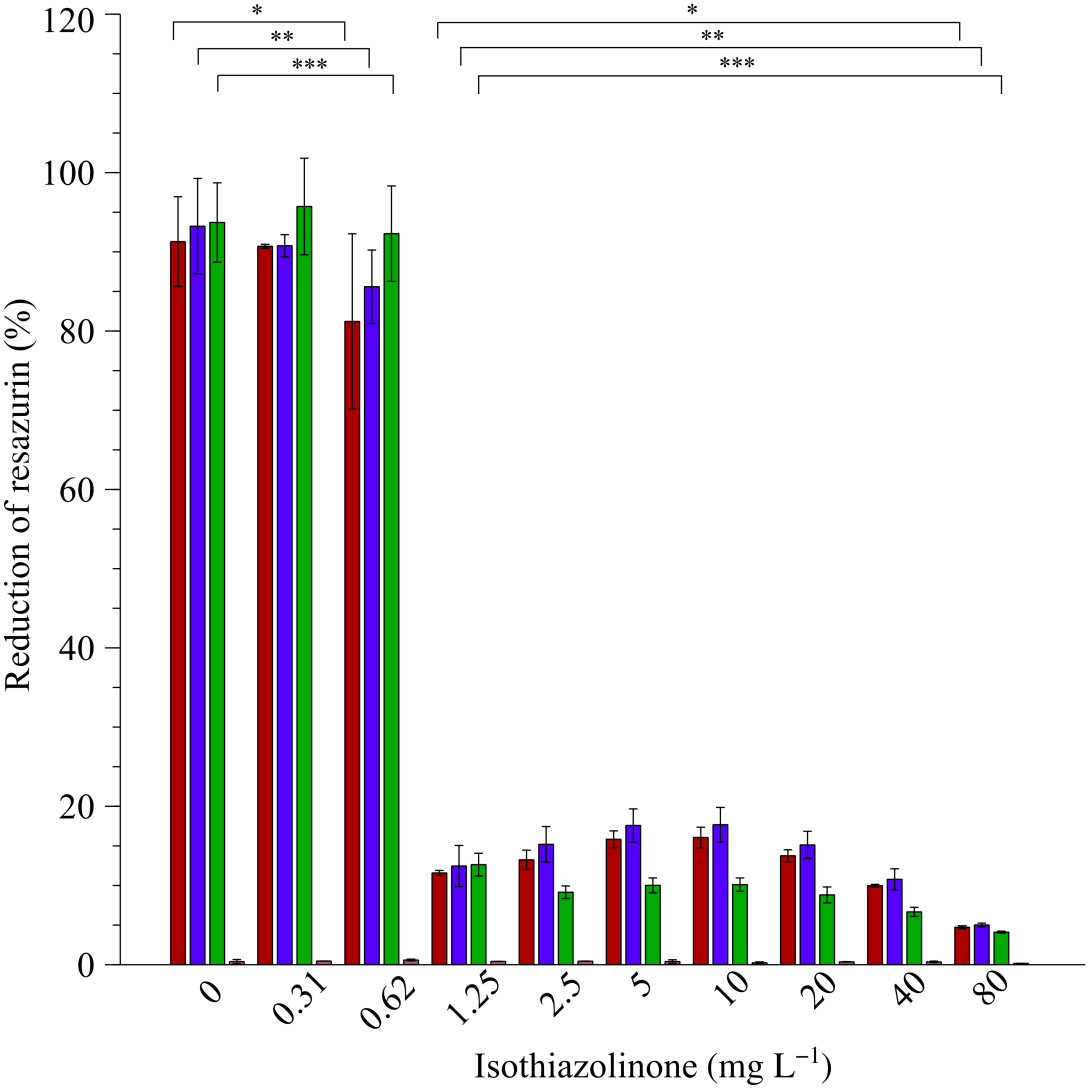
Minimal inhibitory concentration determination of the isothiazolinone‐based biocide using the viability indicator resazurin. The respective percentages of reduced resazurin, as a function of increasing biocide concentrations, are indicated for *Pseudomonas aeruginosa* PA01 (red bars), *Pseudomonas fluorescens* CT07 (blue bars), mixed‐community sump culture (green bars) and the uninoculated, negative control wells containing only biocide and growth medium (pink bars). Each bar represents the average of three replicates of two independent experiments and error bars indicate the standard deviation (*n* = 6). Significant differences between the concentrations for each organism are indicated *P. aeruginosa* PA01 (*), *P. fluorescens* CT07 (**) and sump (***) as determined by analysis of variance (*p* > 0.05).

No significant difference in the percentage of resazurin reduction was observed between 0 mg L^−1^ (growth control) and the two lowest biocide concentrations (0.31 and 0.62 mg L^−1^, respectively) for *P. aeruginosa* PAO1, *P. fluorescens* CT07 and the sump microbial community (Figure 2); on average, 91 ± 4.5% of the resazurin was reduced by all the microorganisms in the presence of these biocide concentrations. The first statistically significant effect on either pure cultures or the community was for biocide concentrations greater than 0.62 mg L^−1^, with a minimum biocide concentration required to inhibit metabolic activity by ~80%, being 1.25 mg L^−1^ in all three cases (Figure 2).

**FIGURE 3:**
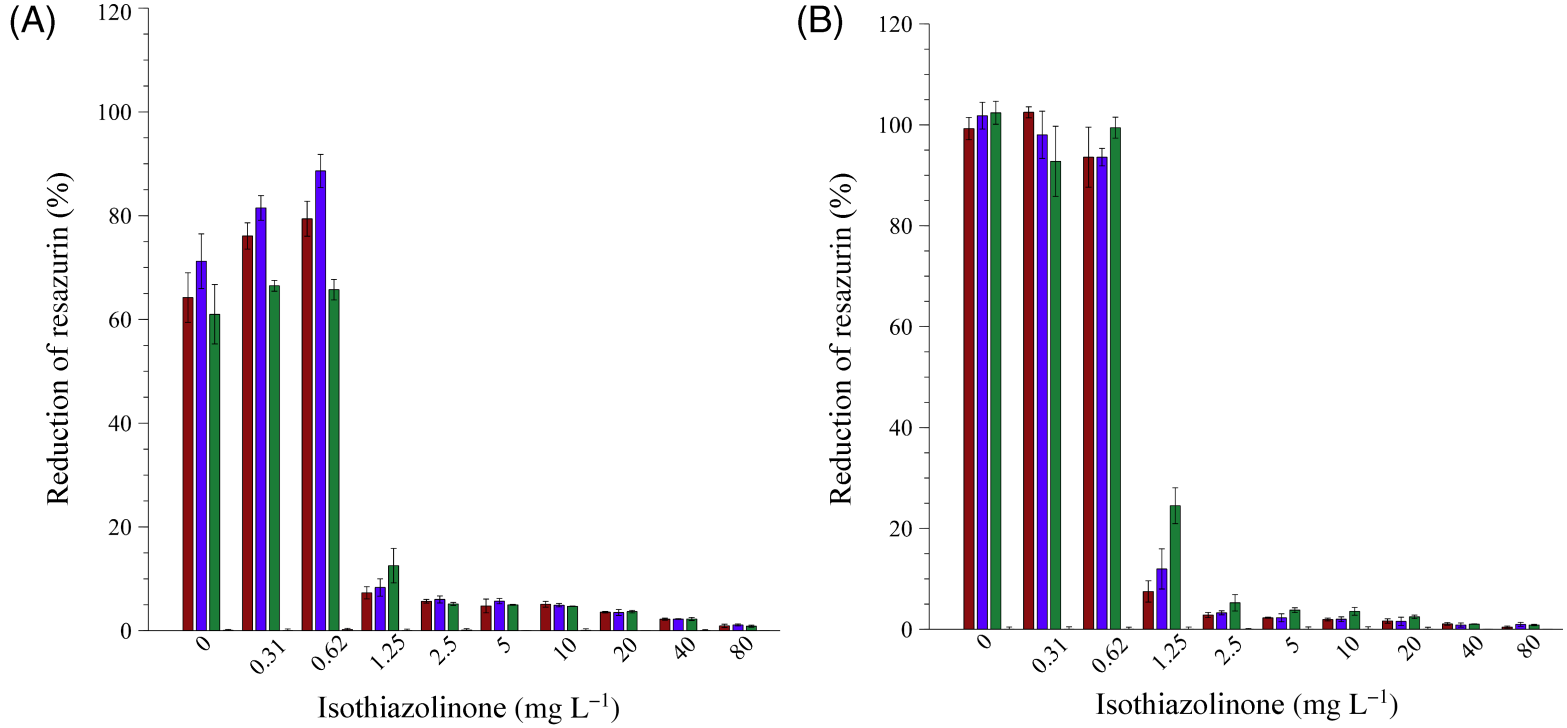
Assessment of the metabolic activity of the (A) planktonic cell populations separated from their (B) sessile counterparts after incubation in the static microplate. The percentage of resazurin reduced is indicative of the metabolic activity of *Pseudomonas aeruginosa* PA01 (red bars), *Pseudomonas fluorescens* CT07 (blue bars), sump microbial community (green bars) and uninoculated mSCW with biocide only (pink bars). Each bar represents the average of three replicates of two independent experiments and error bars indicate the standard deviation (*n* = 6).

**FIGURE A1:**
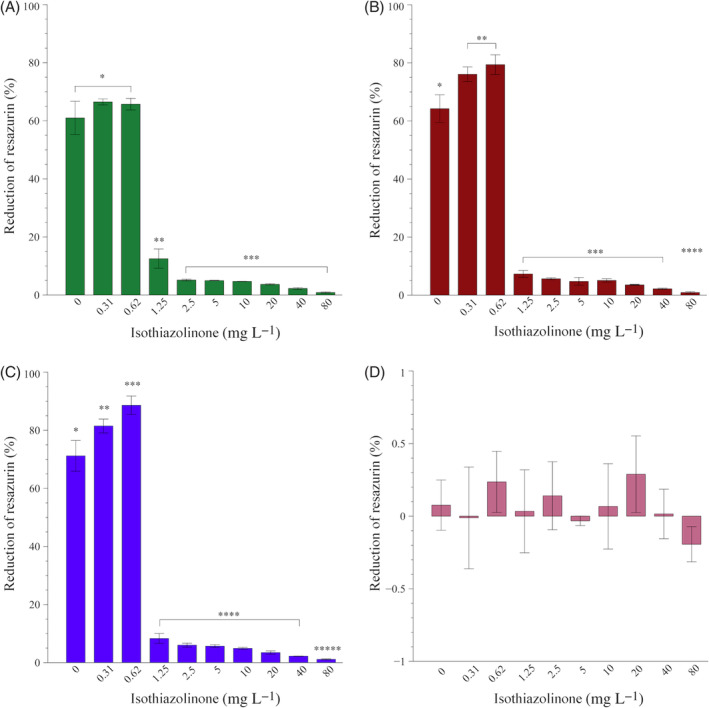
Assessment of the metabolic activity of the planktonic cell populations separated from their sessile counterparts after incubation in the static microplate in the presence of increasing concentrations of isothiazolinone‐based biocide. The percentage of resazurin reduced is indicative of the metabolic activity of (A) sump microbial community, (B) *Pseudomonas aeruginosa* PA01, (C) *Pseudomonas fluorescens* CT07 and (D) uninoculated mSCW with biocide only. Each bar represents the average of three replicates of two independent experiments and error bars indicate the standard deviation (*n* = 6). Significant differences are indicated across the concentration ranges for each organism as indicated by * as determined by analysis of variance (*p* > 0.05).

**FIGURE A2:**
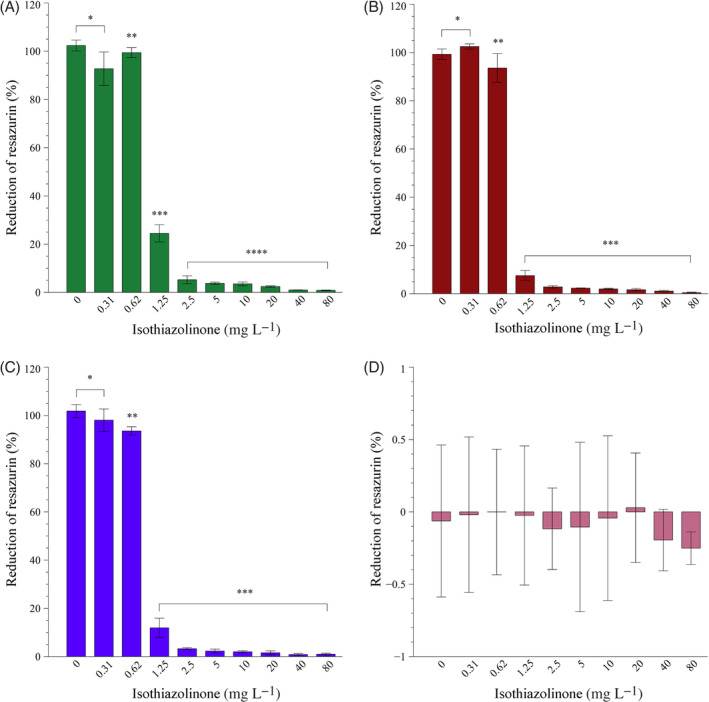
Assessment of the metabolic activity of the sessile populations separated from their planktonic counterparts after incubation in the static microplate in the presence of increasing concentrations of isothiazolinone‐based biocide. The percentage of resazurin reduced is indicative of the metabolic activity of (A) sump microbial community, (B) *Pseudomonas aeruginosa* PA01, (C) *Pseudomonas fluorescens* CT07 and (D) uninoculated mSCW with biocide only. Each bar represents the average of three replicates of two independent experiments and error bars indicate the standard deviation (*n* = 6). Significant differences are indicated across the concentration ranges for each organism as indicated by * as determined by analysis of variance (*p* > 0.05).

The wells containing biocide and growth medium only (uninoculated negative controls) had no influence on the reduction of resazurin in the assay, with less than 1% of resazurin reduced for all the biocide concentrations tested (Figures 2, 3, A1 and A2D, pink bars). This indicated that the reduction of resazurin in inoculated wells is due to microbial metabolic activity.

The extent of resazurin reduction by the planktonic populations exposed to a range of isothiazolinone biocide concentrations (Figure 3A) was similar to the RMIC assay (Figure 2); the metabolic activity of the planktonic populations was unaffected by the two lowest concentrations of the biocide (0.31 and 0.62 mg L^−1^), whereas 1.25 mg L^−1^ of biocide resulted in a significant decrease in activity for the single species as well as the microbial community (Figure A1A–C). The average loss in activity between the sub‐MIC (<1.25 mg L^−1^) and MIC of biocide (≥1.25 mg L^−1^) for all of the microbes was 68 ± 11%. The minimum concentration of biocide required to inhibit the activity of a planktonic population, therefore, corresponded to the MIC determined for the combined planktonic and biofilm populations in the RMIC assay (Figure 2).

A similar trend was observed for the biofilm populations exposed to the sub‐MIC concentrations of the biocide (0.31 and 0.62 mg L^−1^), which had no inhibitory effect on metabolic activity (Figure 3B). However, a statistically significant increase in the activity of the surface‐attached sump microbial community after exposure to the MIC of 1.25 mg L^−1^ was evident, compared to exposures at 2.5 mg L^−1^ or higher (Figure A2A). The activity of the pure culture biofilms exhibited a reduction of 86% (half MIC, 0.63 mg L^−1^) and 82% (MIC, 1.25 mg L^−1^), respectively, whereas biofilms of the microbial community were reduced by 75% for the same concentrations (Figure A2B,C). This suggested that biofilm biomass accumulated in the presence of the MIC of the biocide, which is surprising given that this concentration inhibited planktonic populations of the sump community. Moreover, the reduction of resazurin is evidence that cells in the biofilm remained sufficiently viable to be metabolically active once the inhibitory effect of the biocide was alleviated. To confirm this hypothesis, the CV microplate biofilm assay was employed.

### Static CV microplate biofilm assays

**FIGURE 4:**
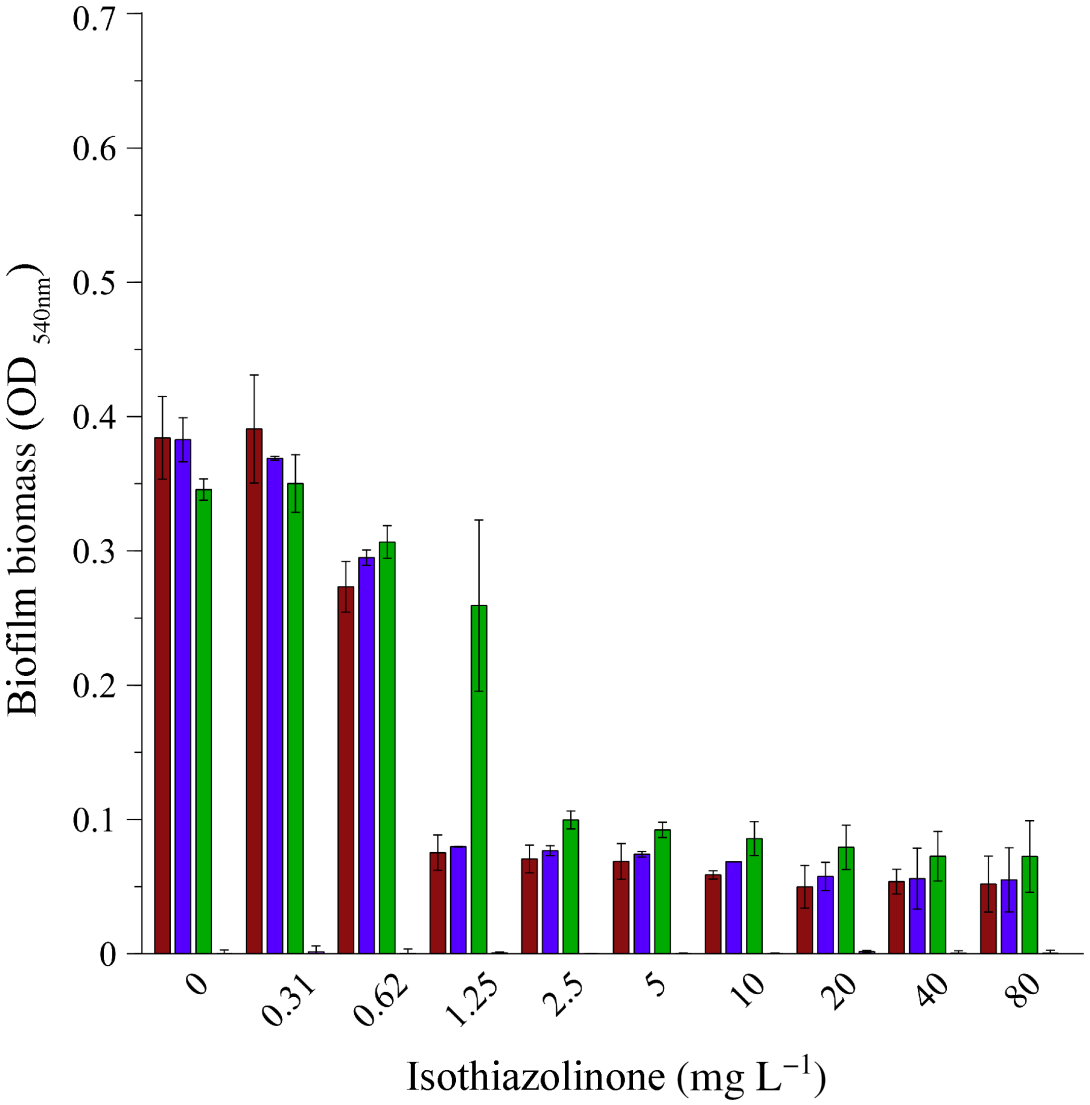
Effect of the isothiazolinone biocide on biofilm biomass accumulation during static incubation in a microplate. Crystal violet‐stained biofilm biomass that developed during 24 h of continuous exposure. Optical density measurements at 540 nm are indicated for *Pseudomonas aeruginosa* PA01 (red bars), *Pseudomonas fluorescens* CT07 (blue bars), sump microbial community (green bars) and uninoculated biocide controls (pink bars). Each bar represents the average of three replicates of two independent experiments and error bars indicate the standard deviation (*n* = 6).

**FIGURE A3:**
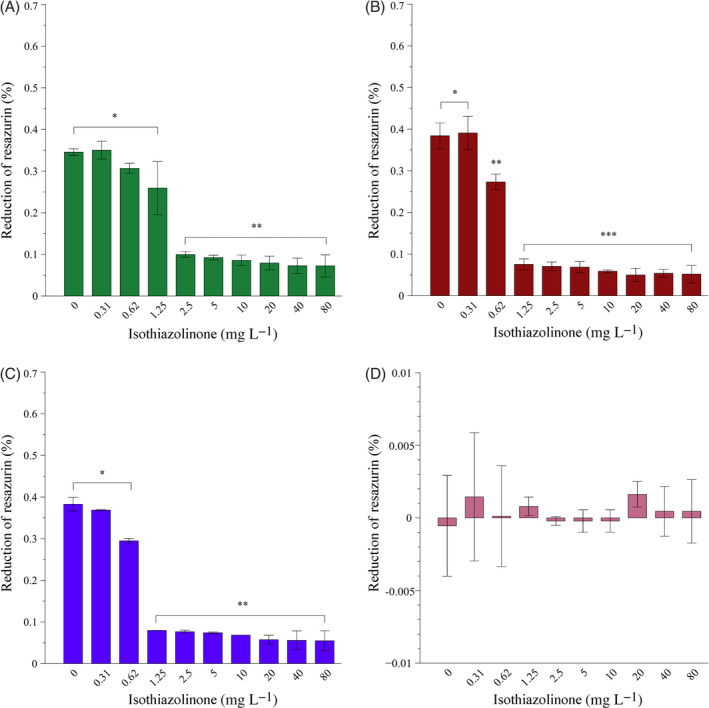
Effect of isothiazolinone biocide on biofilm biomass accumulation during a 24‐h static incubation in a microplate. Crystal violet‐stained biofilm biomass was measured using optical density at 540 nm for (A) sump microbial community, (B) *Pseudomonas aeruginosa* PA01, (C) *Pseudomonas fluorescens* CT07 and (D) uninoculated biocide controls. Each bar represents the average of three replicates of two independent experiments and error bars indicate the standard deviation (*n* = 6). Significant differences are indicated across the concentration ranges for each organism as indicated by * as determined by analysis of variance (*p* > 0.05).

The amount of CV‐stained biofilm biomass attached to the surface of microplate wells after 24 h of incubation in the presence of the biocide, reached similar optical density values for all of the microorganisms in the absence of biocide (0 mg L^−1^) as well as the presence of 0.32 mg L^−1^ of biocide (¼ MIC, Figure 4). A 20% reduction in adhered biomass was observed at a biocide concentration of 0.62 mg L^−1^ for both the single species as well as the sump community, but this decrease was not statistically significant (Figure A3A–C). The inclusion of the biocide at the MIC (1.25 mg L^−1^) significantly reduced the adhered biomass in the wells of both pure cultures (>75% reduction), but not that of the sump microbial community biofilm (Figure A3A–C).

The MIC only reduced the sump community biofilm adherence by 24% compared to 0 mg L^−1^ (OD_540nm_ of 0.34 reduced to 0.26) and no statistically significant difference was observed across the 0–1.25 mg L^−1^ concentration range for the sump community (Figure A3A). This confirmed that the microbial community was capable of biofilm formation in the presence of the biocide MIC. A significant reduction in community biofilm biomass was only observed at concentrations greater than the MIC, with >70% reduction (OD_540nm_ of 0.34 reduced to 0.08 ± 0.01) evident for the 2.5–80 mg L^−1^ biocide concentration range (Figure A3A).

**FIGURE 5:**
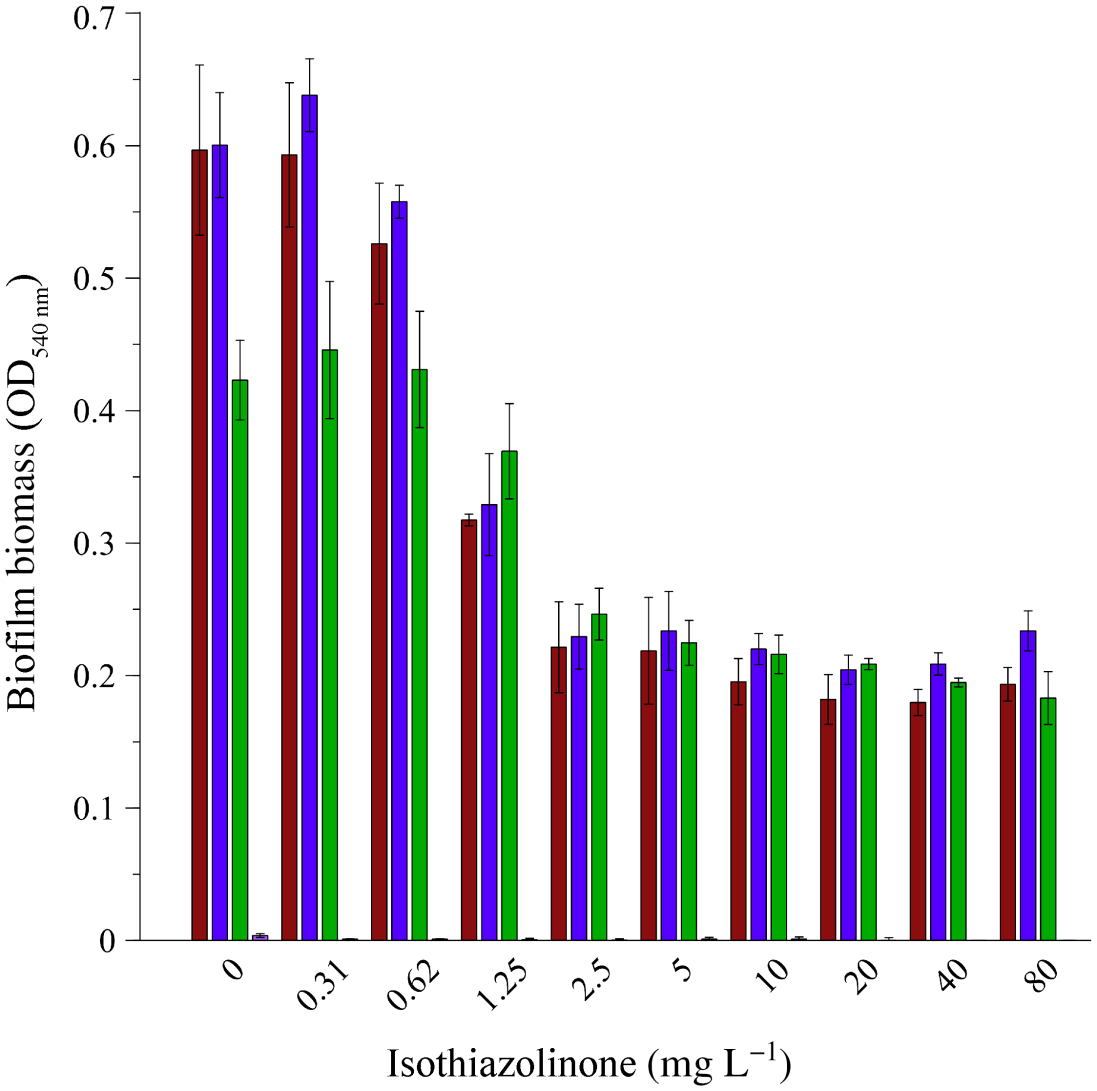
Effect of the isothiazolinone biocide on biofilm biomass removal during static incubation in a microplate. The removal of 24 h‐old biofilms incubated in static microplates was assessed following biocide exposure for an additional 24 h using crystal violet staining of *Pseudomonas aeruginosa* PA01 (red bars), *Pseudomonas fluorescens* CT07 (blue bars), sump microbial community (green bars) and uninoculated biocide controls (pink bars). Each bar represents the average of three replicates of two independent experiments and error bars indicate the standard deviation (*n* = 6).

**FIGURE A4:**
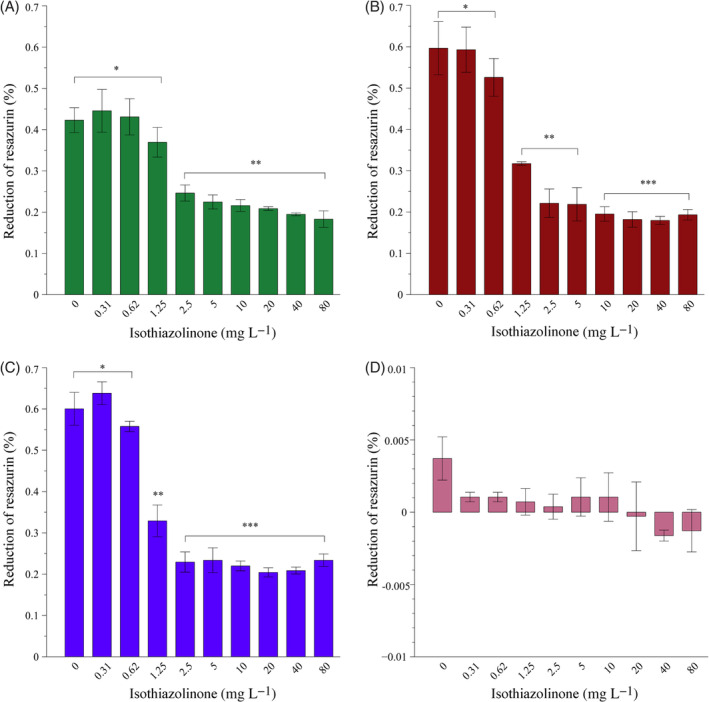
Removal of established biofilm biomass by the isothiazolinone‐based biocide. The removal of 24‐h‐old biofilms incubated in static microplates was assessed following biocide exposure for an additional 24 h using crystal violet staining (A) sump microbial community, (B) *Pseudomonas aeruginosa* PA01, (C) *Pseudomonas fluorescens* CT07 and (D) uninoculated biocide controls. Each bar represents the average of three replicates of two independent experiments and error bars indicate the standard deviation (*n* = 6). Significant differences are indicated across the concentration ranges for each organism as indicated by * as determined by analysis of variance (*p* > 0.05).

The ability of the biocide to remove existing biofilm biomass was also assessed. No significant removal/detachment of biofilm biomass of the single *Pseudomonas* spp. (Figures 5 and A4B,C) occurred in the wells exposed to sub‐MIC of biocide (<1.25 mg L^−1^), with a reduction in biomass observed at the MIC, followed by another decrease and subsequent retention of some biomass at concentrations ≥2.5 mg L^−1^. In contrast, the microbial community (Figure 5, green bars) exhibited no significant removal of biomass for sub‐MIC as well as the MIC of 1.25 mg L^−1^ (Figure A4A). A significant loss of community biofilm biomass (30%) only occurred at concentrations ≥2.5 mg L^−1^ (Figure A4A).

A minimal amount of CV bound to the abiotic surfaces of the wells containing biocide and growth medium only (uninoculated negative controls, less than 4% in Figures A3D and A4D), which indicated that the observed increase in bound dye was due to microbial colonisation of the well surfaces.

### Biofilm‐derived planktonic cell yield and in situ biofilm biomass and metabolic activity monitoring during continuous exposure to various biocide concentrations

The ability of the biofilms to accumulate on the surface of the silicone tubing in the presence of various sub‐MIC concentrations of the isothiazolinone biocide under continuous flow conditions was monitored using the CEMS‐BioSpec system. Changes in the metabolic activity and biomass of the sump community biofilm were determined in real‐time, in conjunction with conventional plate counts of the effluent to determine planktonic cell production by the biofilm. Since the rate at which the growth medium (with and without biocide) was supplied to the CEMS‐BioSpec system displaced the contents of the system every 15 min, it is unlikely that a planktonic population could be maintained in the system (μMax of 0.35–1.01 h^−1^ for *Pseudomonads*, E. Bester unpublished; Ghadakpour et al., 2014; LaBauve & Wargo, 2012; Steiner et al., 2020). It is, therefore, unlikely that an independently replicating population of planktonic cells would persist in the system and it is, therefore, assumed that the planktonic cells enumerated from the system effluent were produced and released by biofilm biomass.

**FIGURE A5:**
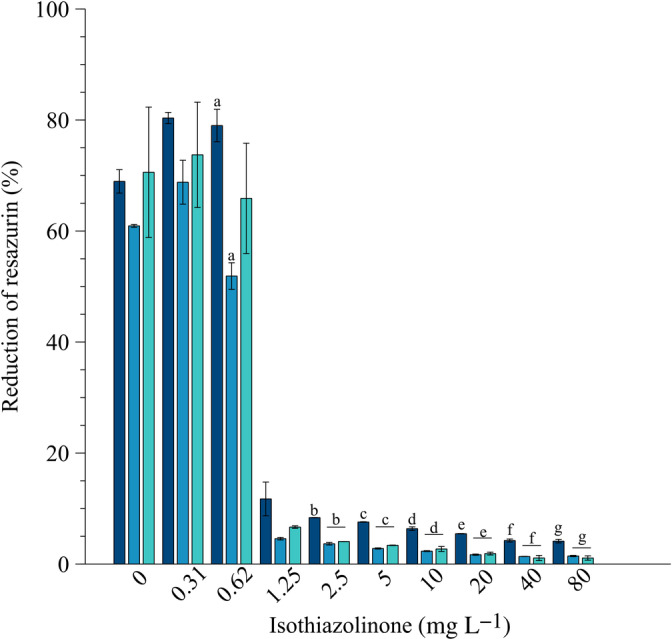
The stability of the isothiazolinone‐based biocide was assessed throughout 120 h. The working stock of the biocide diluted in mSCW was used for RMIC assays 24 h (dark blue bar), 72 h (medium blue bar) and 96 h (light blue bar) after preparation. Each bar represents the average of three replicates of two independent experiments and error bars indicate the standard deviation (*n* = 6). Significance indicated by pair letters as determined (*p* < 0.05).

Before commencing with the continuous exposure experiments, the stability of the isothiazolinone‐based biocide diluted in mSCW was assessed throughout 96 h. While the thermal and pH stability of isothiazolinone‐based biocides is well known, with general stability of 365 days at 25°C and a half‐life of 47 days at a pH value of 8.5 (Barman, 1994; Barman & Preston, 1992; Cloete et al., 1998; Eakins, 2020), the maintenance of activity was determined under the present experimental conditions. An initial loss in activity was observed after 24 h (Figure A5), but this decrease was not statistically significant (*p* > 0.05). After the initial reduction, activity was sustained for the duration of the test period (Figure A5).

**FIGURE A6:**
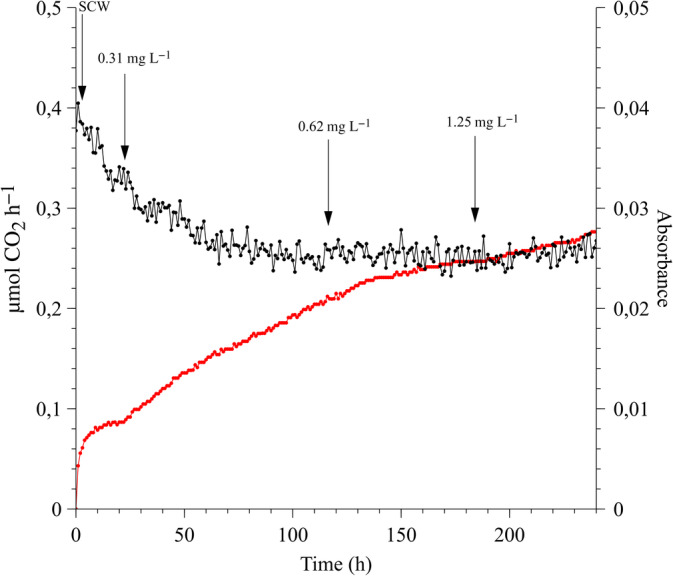
The contribution of abiotic factors to measurements by the CEMS‐BioSpec system. The effects of the sequential introduction of modified synthetic cooling water (mSCW) and increasing concentrations of the isothiazolinone biocide (diluted in the mSCW) on CO_2_ evolution rates (black line) and absorbance measurements (red line) were assessed.

The effect of the isothiazolinone biocide in mSCW on an uninoculated CEMS‐BioSpec system was determined to account for any abiotic contribution to CO_2_ evolution or absorbance measurements. The results indicated that abiotic factors did not contribute meaningfully to either absorbance measurements or CO_2_ evolution rates detected by the CEMS‐BioSpec system (Figure A6).

**FIGURE 6:**
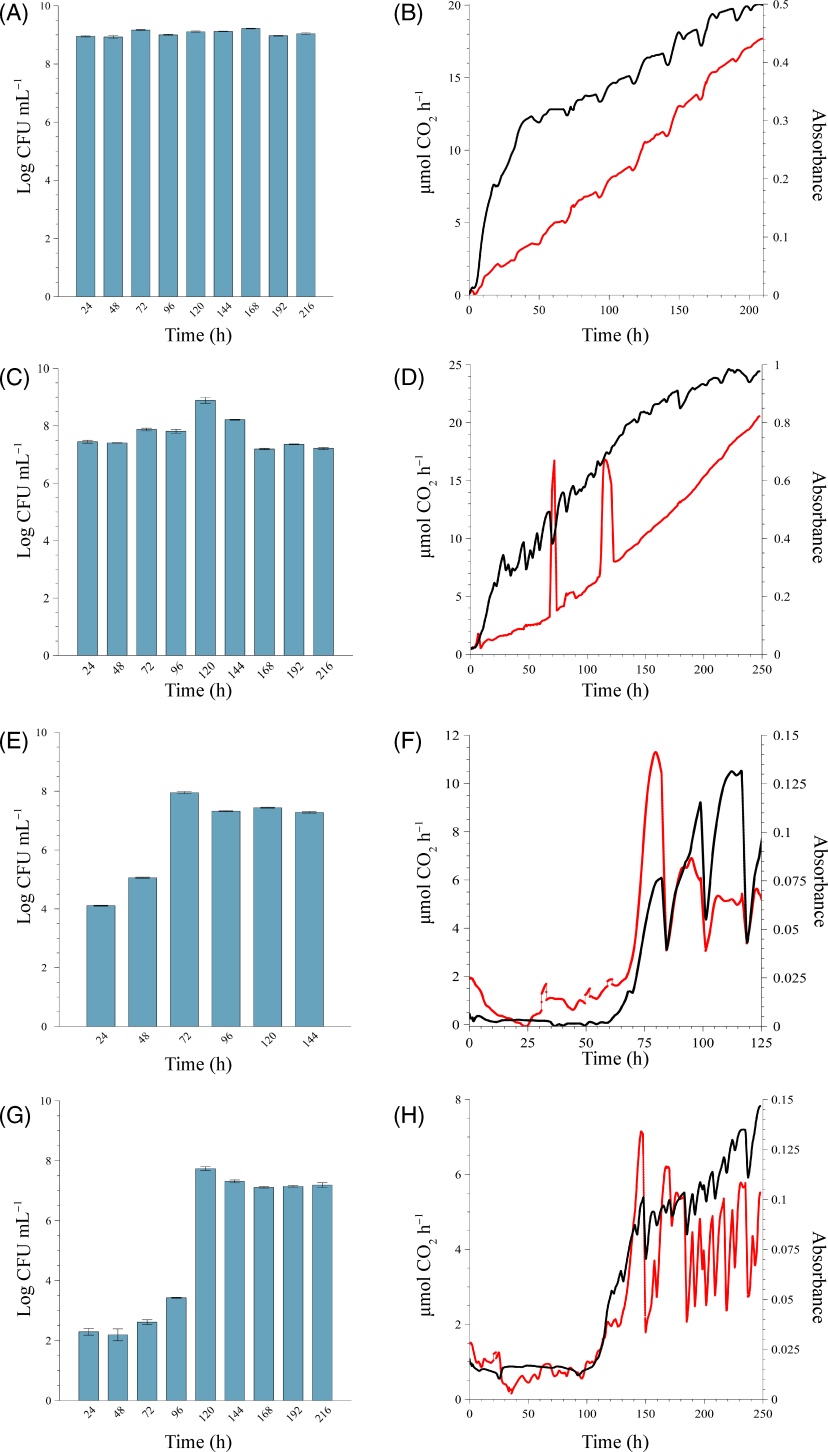
Biofilm formation of the sump microbial community cultivated in the CEMS‐BioSpec system under continuous flow conditions, with modified synthetic cooling water (mSCW) alone or in combination with various concentrations of the isothiazolinone biocide. The concentration of biofilm‐derived planktonic cells in the system's effluent was assessed at 24‐h intervals (A, C, E and G), whereas the changes in biofilm metabolic activity (black line) and biofilm biomass (red line) were monitored in real‐time (B, D, F and H). The concentration of the isothiazolinone biocide in the mSCW was (A and B) 0 mg L^−1^, (C and D) 0.31 mg L^−1^ (quarter MIC), (E and F) 0.62 mg L^−1^ (half MIC) and (G and H) 1.25 mg L^−1^ (MIC). All biofilms were cultivated at a flow rate of 12.5 mL h^−1^ at 22°C and replicate biofilm experiments were conducted for each condition. For clarity, the results of only one experimental repeat per condition are shown here, but the trends for all replicates were similar. Each bar represents the logged mean of triple plate counts, with the error bar representing the standard deviation.

*Growth medium only (0 mg L* ^*−1*^ *biocide)*. The concentration of planktonic cells produced and released by the biofilm was stable throughout the experiment, with an average planktonic yield of log 9.05 ± log 0.1 CFU mL^−1^ (Figure 6A). Biofilm development in mSCW was evident within 5 h after inoculation as an increase in CO_2_ production, followed shortly afterwards by an increase in absorbance due to biomass accumulation (Figure 6B, black and red lines, respectively). In contrast to the remarkable stability in planktonic cell production by the biofilm, a constant increase in both biofilm activity and biomass was observed. A steady state was not achieved for either of these parameters during the experimental period, and the periodic peaks and troughs in both parameters are likely the result of minor sloughing events (Figure 6B).

*Growth medium with ¼ MIC (0.31 mg L* ^*−1*^ *biocide)*. The inclusion of a sub‐inhibitory biocide concentration in the growth medium reservoir resulted in an average planktonic cell yield by the biofilm of log 7.56 ± log 0.36 CFU mL^−1^ (Figure 6C), which was lower than that of the unchallenged biofilm (Figure 6A). Planktonic cell production also remained consistent during biofilm development, with the temporary increase to log 8.89 CFU mL^−1^ at 120 h likely due to effluent sampling coinciding with a biofilm sloughing event (Figure 6D, spike in red line between 110 and 120 h), which would contribute to an increase in effluent cell numbers. The presence of the biocide had a negligible influence on the development of the biofilm (Figure 6D), with an overall increasing trend in both activity and biomass, like that of the unchallenged control biofilm (Figure 6).

The biocide did, however, influence biofilm stability with more frequent and substantive oscillations (peaks and troughs) evident in the metabolic activity (Figure 6D, black line). The amount of attached biomass was generally more stable, with larger peaks evident at both 68 and 110 h (Figure 6B, red line), which coincided with a decrease in metabolic activity, possibly due to the loss of biofilm biomass and a temporary increase in effluent cell numbers, as mentioned previously. While the biofilm did not reach a steady state within the experimental period, the maximum CO_2_ production rate and absorbance values reached were 24 μmol CO_2_ h^−1^ and 0.8, respectively, at 240 h. This was not notably different from that of the unexposed biofilm with corresponding maximum values of 20 μmol CO_2_ h^−1^ at 200 h, but a notable difference was seen in the values with an absorbance of 0.45 at 200 h.

*Growth medium with ½ MIC (0.62 mg L* ^*−1*^ *biocide)*. The inclusion of this concentration of biocide in the mSCW resulted in a pronounced reduction in planktonic cell yield and biofilm development (Figure 6E,F). The presence and subsequent increase in effluent planktonic cell numbers from 24 h (log 4.11 CFU mL^−1^) to 48 h (log 5.06 CFU mL^−1^) confirmed biofilm development in the system before being able to detect its activity. Biofilm biomass increased 10‐fold after 24 h (0.0015–0.0135 absorbance) and metabolic activity was detected after 60 h. A sigmoidal increase in both parameters followed but was not maintained due to significant periodic sloughing events occurring after 75 h (Figure 6F). Once biofilm metabolic activity became detectable (post 60 h), the planktonic cell yield changed less than an order of magnitude (log 7.57 ± log 0.3 CFU mL^−1^) and the same yield was maintained for the duration of the experiment (72–114 h) despite the extensive sloughing events (Figure 6E). The average planktonic cell yield from the biofilm for the duration of the experiment was lower at log 6.38 ± log 1.69 CFU mL^−1^, with the maximum CO_2_ production rate and absorbance value reached at 11 μmol CO_2_ h^−1^ and 0.13, respectively.

*Growth medium with MIC (1.25 mg L* ^*−1*^ *biocide)*. The trends observed for the sub‐MIC biocide concentrations were amplified for biofilms that developed in the presence of the MIC. The initial planktonic cell yields were low (log 2.29 and 2.19 CFU mL^−1^) and only increased after 72 h (Figure 6G). The productivity increased until the biofilm was established (post 150 h), where it stabilised at log 7.15 ± 0.04. The average planktonic cell yield from the biofilms for the duration of the experiment was log 5.22 ± log 2.49 CFU mL^−1^. The MIC delayed initial biofilm establishment by an additional 40 h (Figure 6H), compared to that of the ½ MIC (Figure 6F). The real‐time measurements of biomass (Figure 6H, red line) responded slightly earlier than the metabolic activity (Figure 6H, black line). Like the previous experimentation, the biofilm biomass and metabolic activity proved to be unstable and oscillated after initial biofilm establishment, whereas planktonic cell yield stabilised (Figure 6G). The maximum CO_2_ production rate and absorbance value reached by the biofilm biomass was 5 μmol CO_2_ h^−1^ and 0.14, respectively.

*Growth medium with double MIC (2.50 mg L* ^*−1*^ *biocide)*. The inclusion of double the MIC had a clear bactericidal effect on the sump community. Neither biofilm parameters nor planktonic cell yield increased throughout the 200‐h incubation period for four independent replicates (data not shown).

## DISCUSSION

The deleterious effects of fouling due to biofilm formation occur in both built and natural environments, with wide‐ranging consequences (Dobosz et al., 2015; Rao, 2015). Mitigation strategies to address the operational and financial consequences of biofouling are largely chemical‐centric, employing biocides and biodispersants as the primary treatment approach. Engineered water systems such as cooling towers are rarely, if ever, colonised by a single genera or species, but rather by a diverse community of microorganisms (Iervolino et al., 2017; Paranjape et al., 2020; Salgar‐Chaparro et al., 2020; Srikanth & Berk, 1993). The importance of using a microbial community, especially those originating from industrial cooling systems, rather than a single strain(s) for the study of biocide efficiency is highlighted by Srikanth and Berk (1993). This is in agreement with the results of the batch experiments described here, where the pure cultures and mixed sump community generally responded to the biocide in similar ways (Figures 2, 3, 4). However, as shown here, there are significant differences in the response of sessile biofilms formed by pure or mixed cultures (Figures 3B and 4A).

The study demonstrated the value of resazurin as an indicator of metabolic activity, allowing high‐throughput and time‐efficient screening of non‐oxidising biocides. Results obtained with this assay show that even though planktonic and sessile populations of the pure culture pseudomonads were inhibited the same way by the same concentration of isothiazolinone, this did not hold for a mixed microbial community (Figure 3A,B). Exposure to the MIC as determined experimentally for the sump microbial community reduced the activity of both populations but was more effective against the planktonic population (90%, Figure 3A, green bars) than the sessile population (79%, Figure 3B, green bars). The physical presence of biofilm biomass at the MIC of the biocide (Figure 4), in conjunction with demonstrated activity, corroborates the fact that the sump microbial community was capable of not only forming a biofilm but also maintaining metabolic activity despite the presence of the biocide, which is in line with previous reports (Cloete et al., 1992; Paranjape et al., 2020; Salgar‐Chaparro et al., 2020; Srikanth & Berk, 1993). This observation corroborates the widely accepted potential for a differential response between the two modes of growth to the same antimicrobial treatment (Barman & Preston, 1992; Hjort et al., 2022; Jackson et al., 2015; Salgar‐Chaparro et al., 2020).

As previously stated, static screening tools offer high‐throughput and efficient evaluation of antimicrobials, but their reliance on end‐point analysis lacks the time‐resolved nuances to adequately investigate the dynamic and stochastic nature of biofilms. In addition, the use of biocides in industrial biofouling mitigation strategies generally does not occur in stagnant (batch) systems, but rather in dynamic, continuous flowing conditions (Flemming, 1991, 2011; Iervolino et al., 2017; Paranjape et al., 2020; Pinel et al., 2020; Rao, 2015). We, therefore, focused on the real‐time investigation of the effect of the isothiazolinone biocide at sub‐MIC and MIC on biofilms cultivated under continuous flow conditions.

The dynamic nature of biofilms exposed to flow is clearly illustrated by the continued increase in activity and biomass of sump community biofilms in the absence of the biocide until frequent sloughing events lead to a quasi‐steady state (Figure 6B). Continual exposure to ¼ MIC of the biocide amplified the severity of the sloughing events and increased overall instability, but did not significantly retard or inhibit biofilm accumulation, activity, or planktonic cell yield.

Analyses of the microbial community cultivated under static conditions in the absence of the biocide, showed a similar response as when exposed to the biocide at quarter or half the MIC for 24 h, with no inhibition of metabolic activity (Figures 2 and 3) or biofilm biomass accumulation (Figure 4) evident in these end‐point analyses. However, real‐time investigation of biofilms exposed to the same biocide concentrations under continuous flow conditions revealed a more nuanced response to the biocide. The presence of log 4.1 CFU mL^−1^ of planktonic cells in the system effluent at 24 h provided indirect evidence of active, surface‐associated cells that did not wash out of in the system (Figure 6E), even though a detectable increase in biofilm biomass and metabolic activity was only evident after >24 h. Although biofilm biomass and activity did show a subsequent exponential increase, regular sloughing events prevented biofilm accumulation and sustained an increase in activity (Figure 6F), which suggests some form of regulation to balance growth (depletion of resources) and long‐term survival.

Batch determinations at the biocide's MIC, showed an 80% inhibition of combined planktonic and biofilm population activity, relative to the no‐biocide control (Figure 2). When these populations were separated from each other, it became evident that the surface‐associated fraction of the population contributed more to the observed activity (Figure 3B), which was also supported by the presence of CV‐stained biofilm biomass (Figure 4). The ability to monitor the effect of the biocide's MIC on biofilm dynamics during the continuous supply of fresh nutrients and oxygen under flow conditions, as opposed to static conditions, again revealed more nuanced trends. Initial planktonic cell yields were maintained at low concentrations (log 2.4 to log 3.5 CFU mL^−1^) for the first 96 h (Figure 6G), with minimal biomass accumulation and no detectable biofilm metabolic activity (Figure 6H). An exponential increase in all parameters followed >96 h, and despite frequent and significant sloughing events occurring during biofilm development, planktonic cell yield stabilised at log 7 CFU mL^−1^ for the duration of the experiment, illustrating the often‐ignored role of biofilms in overall proliferation and source of planktonic cells, instead of a primary role of survival. The exposure to double the MIC had the most pronounced effect, with no biofilm formation occurring despite continuous incubation for 200 h.

Microbial biofilms are generally considered to be more resilient to the effects of biocides than their planktonic counterparts. This is due to the inherent properties of biofilms, such as poor diffusion through EPS, altered physiology, heterogenicity, and variable growth rates (Hjort et al., 2022; Jackson et al., 2015). These properties have a direct influence on MIC determinations of biocides, with the planktonic populations having a lower MIC than that of their sessile counterparts (Hjort et al., 2022; Salgar‐Chaparro et al., 2020). Furthermore, even if biocides are dosed at or above the MIC for the particular biocide, the complex, dynamic, and stochastic nature of biofilms often leads to a sub‐lethal dose effect of the biocide (Andersson & Hughes, 2014; Hjort et al., 2022; Salgar‐Chaparro et al., 2020).

The determination of an enhanced MIC under static conditions in microplates through the inclusion of the metabolic activity indicator resazurin provides a valuable tool for high‐throughput, time‐efficient and accurate screening of existing and new non‐oxidising biocides. The separation of sessile populations from their planktonic counterparts may provide further insights into which mode of growth contributes more to the observed recalcitrance to antimicrobial treatment. However, given the dynamic nature of biofilms, it is evident that multifaceted approaches, including real‐time analyses under environmentally relevant conditions, are required to better describe their behaviour when challenged with antimicrobials, also at sub‐MIC ranges to gain improved insights into the mechanisms of biofilm resistance or tolerance.

## CONCLUSIONS

The screening and evaluation of biocides for use in the control of biofouling within an industrial setting is as nuanced and complex as the biofilms they are designed to treat. A holistic approach is required by incorporating various techniques for studying biofilms. The nature of static MIC broth microdilution and CV biofilm assays facilitates high‐throughput screening but cannot inform on the complexity of biofilms that develop under dynamic, continuous flow conditions. A holistic, ‘best of both worlds’ approach would include using the high‐throughput, scalable, simple, cost‐effective screening tool provided by static assays in concert with the more time‐consuming, complex, but more realistic results obtained from continuous flow methodologies. Herein, we propose developing a biocide evaluation pipeline that incorporates the best of both static and dynamic biofilm systems for evaluating the use of a conventional and widely used industrial biocide (isothiazolinone). This approach can yield valuable insights into the development/evaluation of new biocide formulations or an understanding of the potential for the development of robust biofilms under conditions where exposure to sub‐MIC may occur (wastewater treatment systems, natural water bodies receiving effluent, etc).

## AUTHOR CONTRIBUTIONS

**Kyle Klopper:** Conceptualization (equal); data curation (equal); formal analysis (lead); investigation (lead); methodology (equal); visualization (lead); writing – original draft (lead). **Elanna Bester:** Conceptualization (equal); data curation (equal); methodology (equal); project administration (equal); visualization (equal); writing – review and editing (equal). **Martha van Schalkwyk:** Conceptualization (equal); writing – review and editing (equal). **Gideon Wolfaardt:** Conceptualization (equal); funding acquisition (lead); methodology (equal); resources (lead); supervision (lead); writing – review and editing (equal).

## CONFLICT OF INTEREST STATEMENT

Martha van Schalkwyk is currently enrolled in an unrelated master's study in the Department of Microbiology at Stellenbosch University while concurrently being employed by a chemical company that is the manufacturer of the isothiazolinone‐based biocide used in this study. However, the company had no input in the conceptualization, methodology, analysis or reporting of the study. All other authors have no conflict of interest to declare.

## Data Availability

The data that supports the findings of this study are available in the article and its Appendix.

## Appendix

The average metabolic activity of the microbial community over the three time points, as the percentage of resazurin reduced, was 67 ± 5% for the growth medium only (0 mg L^−1^) and 74 ± 6% for the wells containing 0.31 mg L^−1^ of biocide. In contrast, a significant increase in the inhibitory effect of the biocide was seen between 24 and 72 h for 0.62 mg L^−1^ (half MIC) but decreased again at 96 h to levels similar to that of the 24 h time point. The MIC of 1.25 mg L^−1^ exhibited a non‐significant increase of 6.1% in the ability of the biocide to inhibit metabolic activity at 72 and 96 h compared with 24 h (11.7% vs. 4.6% and 6.7%). The inhibitory effect of the biocide at higher concentrations (2.5–80 mg L^−1^) increased significantly from 24 to 72 h and 96 h (activity increased over time by <5%, Figure A1). The results indicate that the biocide retained activity for a minimum of 120 h after dilution in a growth medium.

The effect of the isothiazolinone biocide alone was examined to account for any contribution to CO_2_ evolution or absorbance measurements that can be attributed to abiotic factors. mSCW was used as a baseline for all of the test fluids (Figure A6). The introduction of mSCW had a minimal effect on either of the measured parameters, with an increase of 14% in absorbance (from 0.0094 to 0.108) and 12% in CO_2_ evolution rates (0.751 to 0.855 μmol CO_2_ h^−1^). The sequential introduction of increasing concentrations of the biocide diluted in mSCW led to a gradual increase in CO_2_ evolution rates, with a maximum of 0.78 μmol CO_2_ h^−1^ evolved from the 80 mg L^−1^ dilution (Figure A6). Since the CO_2_ production rates of established sump community biofilms were 10‐fold higher, the abiotic contribution to measured values is therefore considered negligible. Absorbance measurements did not follow the same trend but fluctuated between 0.001 and 0.03, which is similarly well below the values detected for biofilm formation. The results show that abiotic factors did not contribute meaningfully to either absorbance measurements or CO_2_ evolution rates detected by the CEMS‐BioSpec system (Figure A6).
